# Experimental Investigation of a Plate–Frame Water Gap Membrane Distillation System for Seawater Desalination

**DOI:** 10.3390/membranes13090804

**Published:** 2023-09-19

**Authors:** Dahiru U. Lawal, Ismail Abdulazeez, Qusay F. Alsalhy, Jamilu Usman, Sani. I. Abba, Ibrahim B. Mansir, Ravishankar Sathyamurthy, Noel Jacob Kaleekkal, Binash Imteyaz

**Affiliations:** 1 Interdisciplinary Research Centre for Membrane and Water Security (IRC-MWS), King Fahd University of Petroleum & Minerals, Dhahran 31261, Saudi Arabia; ismail.abdulazeez@kfupm.edu.sa (I.A.); jamilu.usman@kfupm.edu.sa (J.U.); sani.abba@kfupm.edu.sa (S.I.A.); 2 Membrane Technology Research Unit, Chemical Engineering Department, University of Technology-Iraq, Alsinaa Street 52, Baghdad 10066, Iraq; qusay.f.abdulhameed@uotechnology.edu.iq; 3 Department of Mechanical Engineering, College of Engineering in Al-Kharj, Prince Sattam bin Abdulaziz University, Al-Kharj 11942, Saudi Arabia; i.balarabe@psau.edu.sa; 4 Centre for Energy Research and Training, Ahmadu Bello University, Zaria P.M.B. 1045, Nigeria; 5 Department of Mechanical Engineering, King Fahd University of Petroleum & Minerals, Dhahran 31261, Saudi Arabia; r.sathyamurthy@kfupm.edu.sa; 6 Interdisciplinary Research Center for Renewable Energy and Power Systems (IRC-REPS), King Fahd University of Petroleum & Minerals, Dhahran 31261, Saudi Arabia; binash.imteyaz@kfupm.edu.sa; 7 Membrane Separation Group, Department of Chemical Engineering, National Institute of Technology (NITC), Calicut 673601, Kerala, India; noel@nitc.ac.in

**Keywords:** hydrophobic, membrane distillation, water access, water poverty, experiment, gained output ratio

## Abstract

This study presented a detailed investigation into the performance of a plate–frame water gap membrane distillation (WGMD) system for the desalination of untreated real seawater. One approach to improving the performance of WGMD is through the proper selection of cooling plate material, which plays a vital role in enhancing the gap vapor condensation process. Hence, the influence of different cooling plate materials was examined and discussed. Furthermore, two different hydrophobic micro-porous polymeric membranes of similar mean pore sizes were utilized in the study. The influence of key operating parameters, including the feed water temperature and flow rate, was examined against the system vapor flux and gained output ratio (GOR). In addition, the used membranes were characterized by means of different techniques in terms of surface morphology, liquid entry pressure, water contact angle, pore size distribution, and porosity. Findings revealed that, at all conditions, the PTFE membrane exhibits superior vapor flux and energy efficiency (GOR), with 9.36% to 14.36% higher flux at a 0.6 to 1.2 L/min feed flow rate when compared to the PVDF membrane. The copper plate, which has the highest thermal conductivity, attained the highest vapor flux, while the acrylic plate, which has an extra-low thermal conductivity, recorded the lowest vapor flux. The increasing order of GOR values for different cooling plates is acrylic < HDPE < copper < aluminum < brass < stainless steel. Results also indicated that increasing the feed temperature increases the vapor flux almost exponentially to a maximum flux value of 30.36 kg/m^2^hr. The system GOR also improves in a decreasing pattern to a maximum value of 0.4049. Moreover, a long-term test showed that the PTFE membrane, which exhibits superior hydrophobicity, registered better salt rejection stability. The use of copper as a cooling plate material for better system performance is recommended, while cooling plate materials with very low thermal conductivities, such as a low thermally conducting polymer, are discouraged.

## 1. Introduction

The demand for freshwater resources is ever-increasing due to the rising global population and agricultural and industrial uses [1]. To meet the needs of freshwater demands, different desalination technologies have been proposed and developed for desalting saline water or seawater, which is available in abundance. For instance, multi-effect distillation (MED) [2,3], multi-stage flash (MSF) [4], reverse osmosis (RO) [5], mechanical vapor compression (MVC) [6], and thermal vapor compression (TVC) [7] have been widely applied for centralized and large-scale freshwater production from salty water. Most of these desalination systems require huge amounts of energy for their operation and are mostly suitable for large-scale applications [8]. Additionally, these desalination technologies require huge capital investments, are complex or complicated, and demand experts or technical know-how for their maintenance and sustainability. In rural or remote settings where there is an abundance of renewable energy, limited expertise or technological know-how, and low-income sources, decentralized and small-scale or medium-scale desalination systems become the most appropriate technology for freshwater production. A membrane distillation (MD) system is one of the desalination techniques that can be deployed for small- to medium-scale freshwater production, especially in rural, remote, and coastal regions. Membrane distillation is an emerging and promising membrane-based thermally driven desalination process that employs a micro-porous hydrophobic membrane for vapor separation from saline water [9,10,11]. Some of the benefits of the MD process, when compared to other desalination techniques, involve low system fabrication costs, low operation and maintenance costs, a high salt rejection factor, flexibility in hybridizing with low-grade and renewable energy sources, flexibility in equipment control, hydrostatic pressure, and compatibility with other membrane-based and thermal-based systems [9,12].

Depending on the approach for vapor condensation, MD can be classified into four major configurations [13], including air gap membrane distillation (AGMD) [14,15,16,17], sweeping-gas membrane distillation (SGMD) [12,18,19,20,21,22], direct contact membrane distillation (DCMD) [12,23,24,25,26,27], and vacuum membrane distillation (VMD) [12,28,29,30,31,32]. Recently, a new MD configuration that combined the features of AGMD and DCMD processes was introduced and named water gap membrane distillation (WGMD) [33,34,35] or permeate gap membrane distillation (PGMD) [36,37,38,39], or liquid gap membrane distillation (LGMD) [40,41,42,43]. A material gap membrane distillation (MGMD) [44,45] and conducting gap membrane distillation (CGMD) [46] have also been disclosed. In an air gap membrane distillation process, thermal efficiency is high due to the creation of an air barrier (air gap) between the permeate side of the membrane and the condensation surface (reducing heat lost by conduction), while mass flux is low in an AGMD system. On the other hand, the DCMD process exhibits higher mass flux but lower thermal efficiency due to the high heat lost by conduction. The water gap membrane distillation process, which improves the weakness of AGMD, exhibits promising results [37,47]. For the WGMD process, the air gap in an AGMD is filled with clean water, such as permeated water, deionized water, or distilled water. Lately, membrane distillation by a water gap is generating a lot of interest from various researchers, investigators, and innovators. For instance, Elbessomy et al. [33] examined the optimum design configurations of a compact water gap membrane distillation hollow fiber module driven by ultra-low waste heat sources. A new fully coupled computational fluid dynamic model was developed and validated against the experimental data and recorded a maximum percentage error of less than 5%. Results indicate an optimum system configuration of 5 cm shell diameter, 10 cm fiber length, packing of 91 fibers, and 1.62 m/s feed stream flow velocity. Furthermore, the system reached a productivity of 12.1 m^3^/day at the optimum configuration, 45 °C feed temperature, and 55 °C coolant temperature. Using composite membranes, a comparison between water gap MD and material gap (different gap materials) MD was made via an experimental and theoretical analysis [35]. Results indicated that using graphite to fill the gap in the material gap MD unit registered about 11 to 22% higher flux than the water gap MD system. However, the water gap MD module recorded about 18 to 27% and 17 to 24% higher flux than the material gap MD when the gap is filled with zeolite and silica gel, respectively. Also, a material with a packing density of 40% attained higher flux than 60% packing density when the material’s thermal conductivity is lower than 5 W/mK and vice versa when the thermal conductivity is greater than 5 W/mK. A water gap MD unit with an internal gap rotating impeller has been proposed and investigated [39,48]. The installed impeller was reported to have improved the heat and mass transfer characteristics of the module, which translates into enhanced vapor flux, better system energy consumption, and improved freshwater cost. Results revealed that the impeller revolution and impeller diameter are the most significant impeller design variables influencing system flux, energy efficiency, and water production cost. On the other hand, the impeller blade material and thickness showed marginal effects on the system’s performance. The performance of a water gap MD system where gap water is externally circulated has also been investigated [49]. Recirculating the water in the gap of the WGMD system improves the flux and reaches a maximum value of about 190 kg/m^2^hr. This also improves the system’s gained output ratio and specific electrical energy consumption in the range of 5 to 22% and 15 to 25%, respectively. Alawad et al. [50] performed a detailed theoretical analysis on a multi-stage water gap membrane distillation unit along with the system economic assessment. Their findings indicated 35 stages as the maximum number of stages and reported a peak freshwater productivity of 5.2 L/h. In the case of an insulated system (insulated module and piping system), the system flux improves by over 35%. Meanwhile, the optimization results indicated an upper limit of 15 stages and a freshwater cost of 3 $m^3^. By using a response surface methodology approach, a modeling and optimization analysis of a commercial spiral wound permeate gap MD system has been conducted by Ruiz-Aguirre [51]. They found the evaporation temperature to be the most dominant parameter affecting the system flux and specific thermal energy consumption (STEC), while the condenser temperature exhibits negligible impact on the system flux and STEC. Increasing the evaporation temperature increases the vapor flux and decreases the system STEC. The optimization results indicate a maximum vapor flux and minimum STEC of 2.66 L/m^2^hr and 255.8 kWh/m^3^, respectively. Through heat and mass transfer analysis, the modeling of hollow fiber permeate gap MD systems has been presented [52]. The model takes into consideration the hollow fiber density and density channel. Their findings showed less effect of cold stream temperature and velocity on the system vapor flux when compared to a DCMD configuration. Also, it was established that the hydrodynamics within the coolant and permeate channels have less impact on flux than the channel thermal conductivity of the gap. Meanwhile, changes in the gap channel of PGMD resulted in a more complex combination. Furthermore, the findings imply that the GOR, which reaches a maximum value of 2.4 with 20 stages, rises with the number of stages.

From the foregoing review and the existing open literature, there is a limited or lack of experimental investigation involving different cooling plate materials and different membrane materials using a flat-sheet plate–frame WGMD module for the desalination of untreated real seawater. A better insight into the impact of different cooling plate materials, which is crucial in the gap condensation phenomenon, will facilitate a better understanding and future scale-up of membrane distillation systems. To the best of the authors’ knowledge, this is the first study to experimentally investigate the effect of different cooling plate materials on the performance of a plate–frame water gap MD unit using different types of polymeric membrane materials. Therefore, this current work is aimed at assessing the impact of different cooling plate materials (copper, aluminum, brass, stainless steel, HDPE, and acrylic) since they play a major role in the heat and mass transfer phenomena in the gap chamber. Furthermore, the study investigates the impact of different membrane materials, including a PTFE membrane and a PVDF membrane, on the system’s performance. The investigated system performance includes vapor flux and gained output ratio (GOR), which are evaluated at different feed stream flow rates and temperatures.

## 2. Materials and Methods

### 2.1. Materials

Two different commercially available flat-sheet hydrophobic membranes were characterized: a PTFE (polytetrafluoroethylene) membrane with polypropylene fibrous support and a PVDF (Polyvinylidene Fluoride) membrane with PET substrate, both supplied by TISCH Scientific, Cleves, OH, USA. Their references are SF17386 for the PTFE membrane and SF17387 for the PVDF membrane, with each membrane corresponding to a mean pore size of 0.45 μm, as indicated by the manufacturer. These membranes were used in this work to examine the impact of different cooling plate materials on the system vapor flux and gained output ratio.

### 2.2. Membrane Characterization

Various characterization approaches were used to determine the membrane’s characteristics. The pore size and pore size distribution of the membranes were measured using a capillary flow porometer (3 Gzh, Quantachrome Instruments, Boynton Beach, FL, USA). The used wetting liquid agent was POREFIL 125, and the membranes were first soaked to fill the pores of membranes with the porofil liquid. The mean size of the pores was determined from the intersection between the half-dry curve representing 50% gas flow through the dry membrane sample and the wet curve (the half-dry curve is the mathematical half of the dry curve) using the Laplace equation, as expressed in Equation (1). The bubble point flow rate for the PTFE and PVDF membranes was 0.0451 L/min and 0.0743 L/min, respectively, while the corresponding bubble point pressure was 1.3099 bar and 1.2654 bar. The porosity (ԑ) of each membrane was evaluated using a gravimetric approach, as reported in [53] at ambient temperature. Before and after being soaked in ethanol solvent, the membranes were weighed. After complete soaking of the membrane, filter papers were used to remove any ethanol solvent residue from the membranes’ surface. The membrane’s liquid entry pressure of water (LEP_w_) was measured using a laboratory-made dead-end LEP apparatus, where DI water was pressurized against one side of the membrane surface until a first drop of water was observed on the other end of the membrane surface [54]. Each membrane’s thickness was measured using an electronic micrometer made by Schut Geometrical Metrology in at least 10 distinct locations. The average values and their standard deviations were then reported. The membrane’s hydrophobicity (water contact angle) was determined at room temperature using a goniometer DM-501 Kyowa Interface Science Co., Ltd., Saitama, Japan, instrument by fixing a piece of membrane sheet on a glass slide. After a droplet of deionized water appeared at five different points on the membrane surface, the average value of the water contact angle (WCA) was calculated and recorded. A source light and a lens were used to create the drop image on a screen, and the WCA was measured with the projected drop image. By placing and depressing the tip of the syringe near the membrane surface to generate a constant water drop volume of 5 μL, the membrane WCA was measured. The digital images of the used membrane surface morphology were obtained by SEM (JEOL, Tokyo, Japan) at 10 kV. To reduce charging and increase image resolution, the DII-29030SCTR smart coater was used to coat all samples with a 2 nm coating of gold.

(1) $$d_{p}=\frac{4\sigma }{\Delta P}\times \cos\theta$$

where σ is the surface tension of the POREFIL 125, ΔP the registered pressure, and θ the contact angle between a drop of POREFIL 125 and the membrane surface.

(2) $$\varepsilon \left(\%\right)=\frac{\frac{w_{a}-w_{b}}{d_{e}}}{\frac{w_{a}-w_{b}}{d_{e}}+\frac{w_{b}}{d_{m}}}$$

where $w_{a},w_{b},d_{e},$ and $d_{m}$ are the wet membrane weight, dry membrane weight, ethanol density (0.789 g/cm^3^), and membrane density (2.20 g/cm^3^ for PTFE and 1.740 g/cm^3^ for PVDF), respectively.

### 2.3. MD Experiment

**Figure 1:**
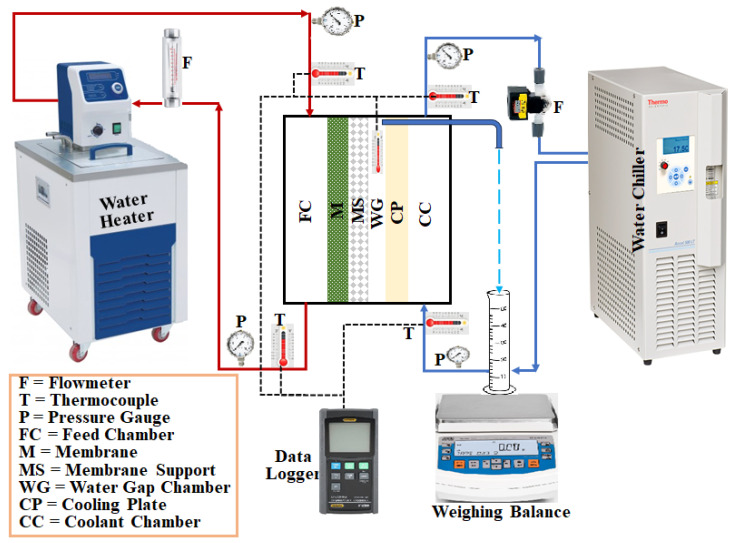
A schematic diagram of the WGMD experimental test rig.

**Figure 2:**
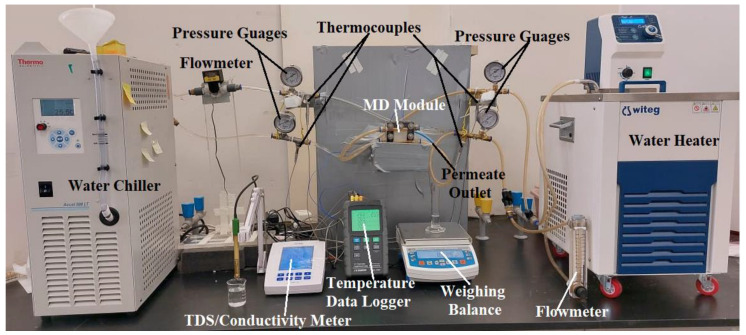
A picture of the WGMD experimental test rig.

**Figure 3:**
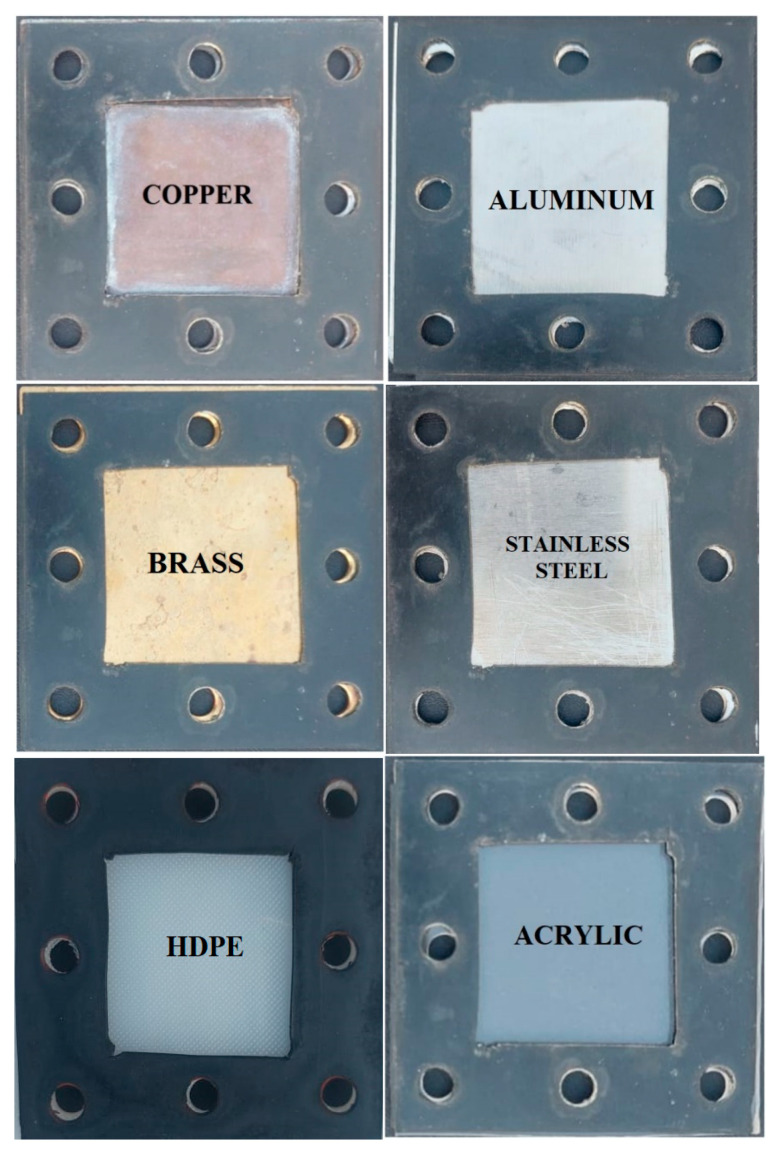
Different cooling plate materials.

Figure 1 shows a schematic illustration of the lab-scale water gap membrane distillation (WGMD) test rig. The set-up’s main components included a WGMD module, a cold stream recirculating bath (Accel 500 LT ThermoFisher Scientific, Waltham, MA, USA), and a hot water recirculating bath (WRC-P8 Witeg, Wertheim, Baden Württemberg, Germany). The membrane module consisted of a membrane sheet, a coolant chamber, a feed chamber, a cooling plate, and a water gap chamber. While the coolant chamber, the feed chamber, and the water gap chamber were made from acrylic material, the cooling plate was made of different materials, including copper plate, aluminum plate, steel plate, brass plate, and acrylic plate. The water gap chamber, the hot chamber, and the cold chamber had dimensions of 40 × 40 × 5 mm each, and a cooling plate of 1.5 mm thick was utilized for heat exchange between the water in the gap chamber and the coolant stream. The actual photograph of the experimental set-up is presented in Figure 2. The membrane was supported and prevented from bulging by a 2 mm perforated HDPE material, which also provided a water gap width of 5 mm between the membrane and the cooling plate. The perforated HDPE material creates an effective membrane area of 7.316 × 10^−4^ m^2^. Figure 3 represents pictures of different cooling plate materials used in this study.

For the WGMD bench-scale tests, the coolant stream was chilled to 15 °C in the recirculating cold bath and supplied to the MD coolant chamber at 2000 mL/min, while the feed saline water was heated to the necessary temperatures (40, 50, 60, and 70 °C) in the recirculating hot water bath and fed to the MD feed chamber at 600, 800, 1000, and 1200 mL/min. Other conditions under which each test was conducted are presented below each figure for convenience. The coolant and feed stream temperatures were measured and recorded by K-type thermocouples and the RDXL-12SD—Omega Data Logger. The coolant and feed stream flow rates were monitored using an FTB336D-PVDF Omega microflow meter and an Omega FL50000 flowmeter, respectively. The produced permeate flux was collected in a graduated cylinder and measured using a Radwag PS 8100.R2.M precision balance. All the WGMD tests were carried out using an untreated real seawater sample with a TDS of 48,200 ppm that was collected from the Arabian Gulf, Saudi Arabia. The feed and permeate salt concentrations were measured by a Hanna HI5321 conductivity/TDS meter. It is worth mentioning that each WGMD experimental data set was carried out for about an hour.

### 2.4. Analytical Methods

The AGMD system’s performance was evaluated in terms of water flux (J_w_) and specific thermal energy consumption (STEC). The new AGMD flux and salt rejection efficiency (SR) were calculated from Equations (3) and (4), respectively.

(3) $$J_{w}=\frac{W}{tA_{em}}$$

(4) $$SR=\frac{C_{f}-C_{p}}{C_{f}}\times 100\%$$

where $J_{w}$ (kg/m^2^.hr), W (kg), t (h), and $A_{em}$ (m^2^) indicate the vapor flux, weight of the collected permeate, duration of distillate collection, and the effective membrane area. The SR (%), $C_{f}$ (g/L), and $C_{p}$ (g/L) are the salt rejection efficiency, the feed concentration, and the permeate concentration.

The system gained output ratio (GOR), which represents the energy efficiency of the system, is represented by [55]:

(5) $$GOR=\frac{J_{w}\times A_{em}\times ∆H_{v}}{3600\times \left({\dot{Q}}_{feed}+{\dot{Q}}_{coolant}\right)}$$

where $∆H_{v},$ $J_{w},\;A_{em}$, ${\dot{Q}}_{feed},and\;{\dot{Q}}_{coolant}$ are the enthalpy of vaporization of the water (kJ/kg), vapor flux (kg/m^2^hr), membrane effective area (m^2^), heat input from water heater (kW), and thermal demand from water chiller (kW), respectively.

## 3. Results

### 3.1. Membrane Characteristics

**Figure 4:**
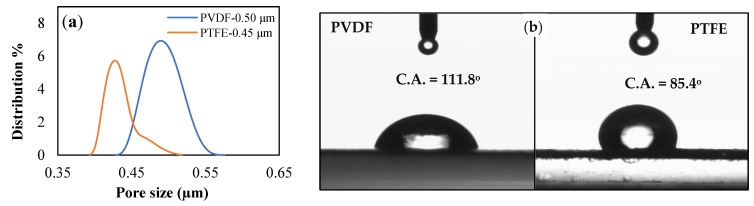
Characteristics of PTFE and PVDF membranes (**a**) Pore size distribution, (**b**) Membrane water contact angles.

**Figure 5:**
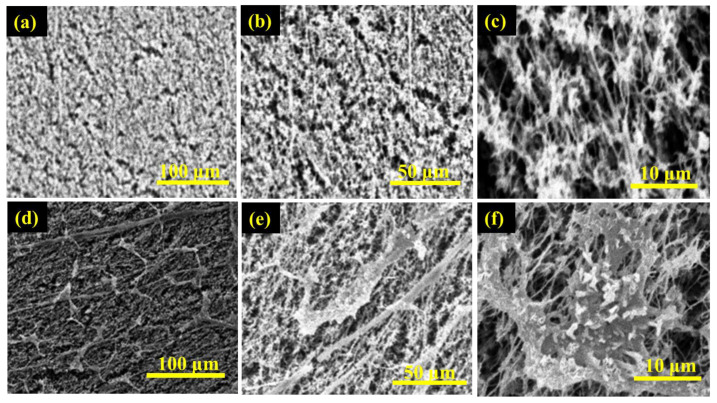
SEM images of the top surface of PTFE membrane (**a**–**c**)—before and (**d**–**f**)—after the desalination of untreated real seawater.

**Figure 6:**
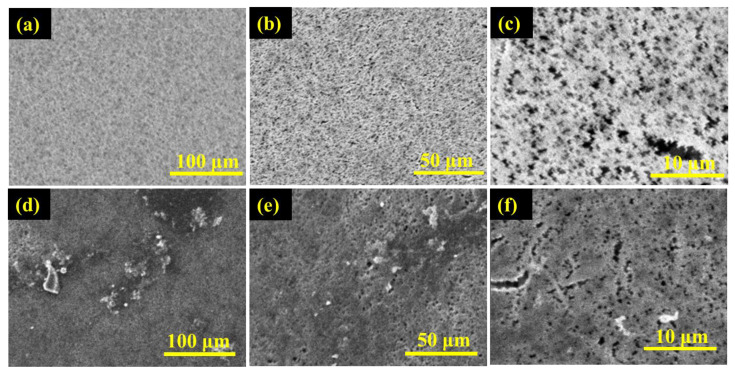
SEM micrographs of the active surface of PVDF membrane (**a**–**c**)—before and (**d**–**f**)—after the desalination of untreated real seawater.

The membrane properties obtained from the membrane characterization are summarized in Table 1. Figure 4a,b displays the obtained membrane’s pore size distributions and membrane water contact angle, respectively, while Figure 5 and Figure 6 show SEM of the membrane surface morphology before and after use in the MD desalination of seawater. The PTFE membrane with its support layer had a total thickness of 156 μm, while the total thickness of the PVDF membrane was 105 μm. The PVDF membrane had a larger mean pore size and a wider pore distribution when compared to the PTFE membrane. Also, the PVDF membrane had a lower contact angle, which may lead to poor wetting resistance, as confirmed by the lower LEP_w_ of the PVDF membrane. The higher hydrophobicity of the PTFE membrane, as revealed by the contact angle in Figure 4, the smaller membrane pore size, and the higher membrane thickness may be responsible for the higher LEPw in the PTFE membrane.

The scanning electron microscope digital images of the surface morphology of PTFE and PVDF membranes at different magnifications are depicted in Figure 5 and Figure 6. Figure 5a–f is the SEM micrographs of the active surface of the PTFE membrane before and after use in the desalination of untreated real seawater having a TDS of 48,200 ppm, while Figure 6a–f is the SEM micrographs of the top surface of the PVDF membrane before and after use in the desalination of untreated real seawater having a TDS of 48,200 ppm. It can be observed that the two membranes showed porous, spongy, and fibrous morphology. Furthermore, some presence or traces of salts (fouling) were noticed on the surface of the used membranes after 15 h in operation for the PTFE membranes and 7 h under test for the PVDF membrane. The longer testing time of the PTFE membranes revealed why more traces of salt crystals and other contaminants were formed on the membrane's active layer when compared to the PVDF membrane.

### 3.2. Influence of Feed Temperature and Cooling Plate Material

**Figure 7:**
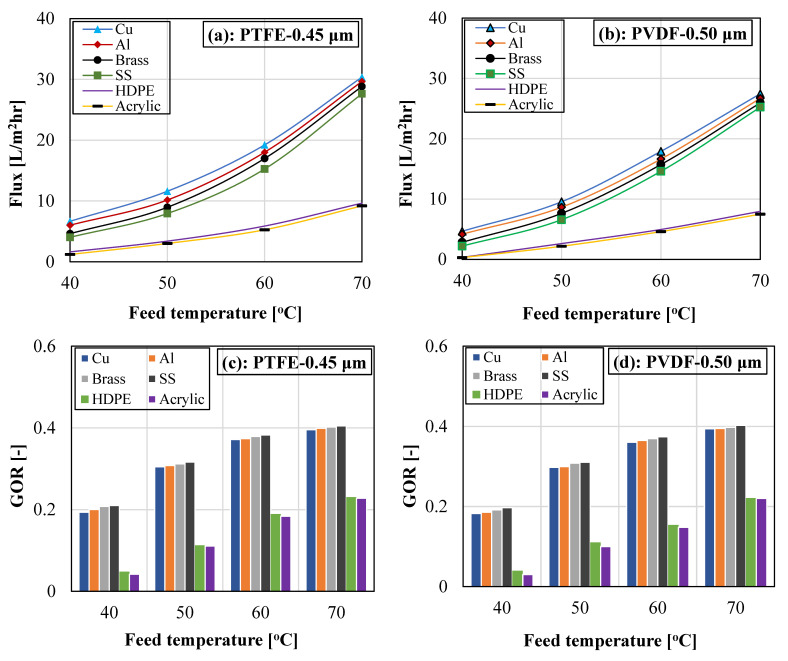
(**a**–**d**). Influence of cooling plate material and feed temperature on vapor flux and GOR for PTFE and PVDF membranes. Test Conditions: Coolant temperature = 15 °C, Coolant flow rate = 2000 mL/min, and Feed flow rate = 1000 mL/min.

The variation in system vapor flux and gained output ratio with different cooling plate materials, including a copper plate, an aluminum plate, a brass plate, a stainless-steel plate, a high-density polyethylene (HDPE) plate, and an acrylic plate, at different feed water temperatures is illustrated in Figure 7 for different membrane materials (PTFE and PVDF membranes). For easy identification, the copper plate, the aluminum plate, the brass plate, the stainless-steel plate, the high-density polyethylene, and the acrylic plate were designated as Cu, Al, brass, SS, HDPE, and acrylic, respectively.

It can be noticed that both the vapor flux and the GOR increase with the rising feed water temperature for each cooling plate and for each membrane material. The vapor flux rises exponentially with the feed water temperature due to the exponential growth in the water vapor pressure with temperature. For instance, increasing the feed temperature from 40 °C to 70 °C for the copper plate enhanced the system vapor flux by over 350% when using a PTFE membrane. The exponential boost in vapor flux is also responsible for the increase in the system GOR with feed temperature. The GOR, as described by Equation (3), was largely controlled by both vapor flux and input energy, with higher vapor flux and lower input energy providing the best GOR. An increase in feed temperature increases the external input energy; however, the effect of the recorded improvement in vapor flux was canceled and exceeded the adverse effect of high input energy. Thus, system GOR improves with feed temperature. For the copper cooling plate, the system GOR improves by over 100% when the feed temperature was elevated from 40 to 70 °C.

For this study, it is anticipated that changing the cooling plate material will have an impact on the overall heat transfer from the cold coolant to the gap water. It can also be observed that the copper plate registered the highest vapor flux, which was closely followed by the aluminum plate, then the brass plate, which was trailed by the stainless-steel plate, followed by the HDPE plate, and lastly, the acrylic plate. The order of the recorded flux is in conformity with the cooling late thermal conductivity, with the cooling plate having the highest thermal conductivity (Cu), attaining the highest flux, and the cooling plate possessing the lowest thermal conductivity (Acrylic) attaining the lowest vapor flux. The thermal conductivity associated with each cooling plate is presented in Table 2 [56,57].

On comparison between the plates with the highest and the lowest thermal conductivity, the copper and the acrylic plates had thermal conductivities of 397 W m^−1^ K^−1^ and 0.20 W m^−1^ K^−1^, respectively, and thicknesses of 2 mm and 2.3 mm, respectively. The sensible conductive heat transfer across a cooling plate is directly proportional to the plate’s thermal conductivity (k) and inversely proportional to the plate thickness (t). Thus, the sensible conductive heat transfer across a plate is related by k/t. Using the corresponding thermal conductivities and thicknesses of Cu and acrylic plates in Table 2, the copper plate recorded over 2000-fold the conductance of the acrylic plate, which showed that the copper plate had a considerably lower thermal resistance than the acrylic plate. This is evident in Table 3, where the mean gap temperature at 70 °C feed temperature was 32.5 °C and 62.2 °C for the copper plate and the acrylic plate, respectively, when using a PTFE membrane. The same findings can be observed in Table 2 for the PVDF membrane at various feed temperatures, which conveys the reason why the vapor flux for the copper plate was considerably higher than that of the acrylic plate.

The copper plate also attained a better GOR than the acrylic plate, even though the temperature drops in the feed and coolant chambers was considerably higher for the copper plate, which indicates higher external input energy for the copper plate. However, the recorded flux in the copper plate greatly exceeded that of the acrylic plate, which consequently resulted in a better GOR for the copper cooling plate. The stainless-steel cooling plate attained a slightly higher GOR than the copper cooling plate due to the larger temperature drops in the feed and coolant chambers of the copper cooling plate, which translates to a higher input energy and, consequently, a lower GOR.

At each test condition, the PTFE membrane was seen to edge over the PVDF membrane in terms of vapor flux and GOR. The PTFE and PVDF membranes attained a flux variation between 6.66 and 30.36 kg/m^2^hr and 4.67 and 27.46 kg/m^2^hr, respectively, when using a copper cooling plate. A corresponding GOR variation of 0.2146–0.4049 and 0.21972–0.4028 was obtained when using stainless-steel cooling plates at 70 °C feed water temperature. Based on Table 1, the PVDF membrane had a slightly larger mean pore size and porosity with a lesser total thickness, which should have supported higher flux. However, the PTFE membrane was a composite membrane with a very thin active layer of around 8 ± 2 μm and a fibrous support layer of about 143 ± 16 μm. The thinner active layer of the PTFE membrane and its higher water contact angle resulted in its superior flux against the PVDF membrane. In addition, the PTFE membrane has a lower surface energy compared to the PVDF membrane [58], which also contributes to more vapor attraction, thereby improving the membrane flux and pore-wetting resistance of the PTFE membrane. The GOR for the PTFE membrane was also higher than the PVDF membrane due to the higher vapor flux, although both had a similar thermal conductivity with values of 0.18–0.22 W/m-K and 0.235–0.2651 W/m-K for PVDF membrane and PTFE membrane, respectively [59,60]. The slightly lower thermal conductivity and thicker active layer thickness of the PVDF membrane supported the lower mean gap temperature reported in Table 3. The PVDF membrane attains a slightly lower gap temperature due to poorer heat conduction from the membrane feed side and across the membrane thickness.

### 3.3. Impact of Feed Flow Rate and Cooling Plate Material

**Figure 8:**
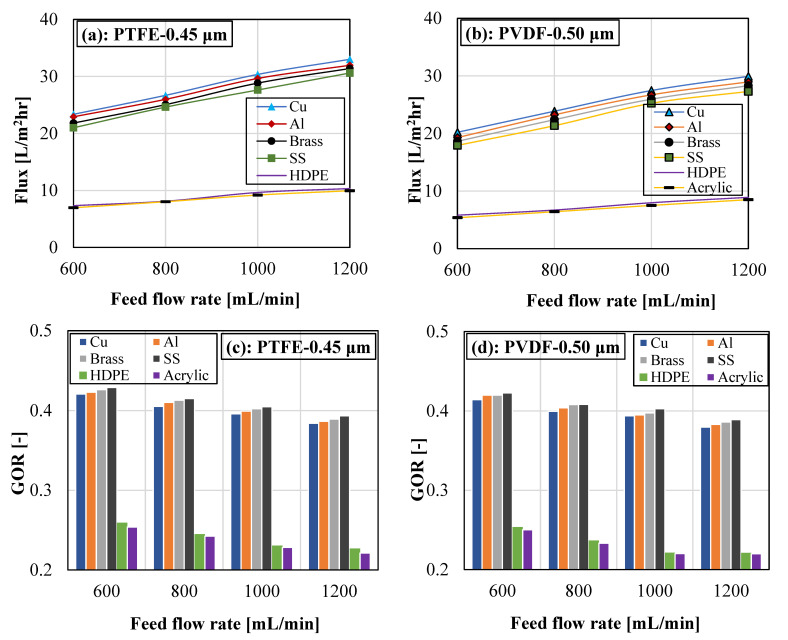
(**a**–**d**). Impact of cooling plate material and feed flow rate on vapor flux and gained output ratio for PTFE and PVDF membranes. Test Conditions: Feed temperature = 70 °C, Coolant temperature = 15 °C, and Coolant flow rate = 2000 mL/min.

The effect of feed-flow rate cooling plate material on the system vapor flux and gained output ratio is demonstrated in Figure 8 for PTFE and PVDF membranes. For both membranes and each cooling plate, increasing the feed flow rate improved the vapor flux and decreased the GOR. Boosting the feed flow rate enhanced the turbulence level on the feed side of the membrane. This reduced the thermal boundary layer thickness on the feed side of the membrane and improved the heat and mass transfer coefficient of the system. Consequently, the vapor flux of the system was improved. Also, increasing the feed flow rate provides a shorter residence time and lessens the heat polarization effect. Figure 8 revealed that GOR decreased with the feed flow rate. The observed reduction in the GOR when increasing the feed flow rate is due to the higher input energy, which is caused by higher heat and mass transfer activities on the feed channel at a higher feed flow rate. Increasing the feed flow rate improves the system productivity; however, the external heat load of the water heater also increases, leading to a decline in the system GOR. The findings in Figure 8a,b revealed that the copper cooling plate yielded the highest flux, while the acrylic cooling plate produced the lowest vapor flux. The variations in the vapor flux for various cooling plates can be attributed to the variations in their thermal conductivity, as discussed in Figure 7.

Figure 8a,b revealed that the stainless-steel plate registered a slightly higher GOR when compared to the rest of the cooling plate materials. The copper cooling plate attained the highest vapor flux; however, the temperature drop in the feed and coolant chambers of the module is also higher for the copper cooling plate. This resulted in higher input energy and, consequently, lower GOR. But in the case of acrylic, its thermal conductivity is extremely low, which leads to its ultra-low vapor flux and resultantly lower GOR. As previously observed in Figure 7 and its comments, the PTFE membrane recorded superior performance (flux and GOR) over the PVDF membrane at various feed water flow rates in Figure 8. For instance, for the copper cooling plate, the PTFE membrane flux ranges between 23.60 and 33.01 kg/m^2^hr, while the corresponding vapor flux for the PVDF membrane varies from 20.21–29.92 kg/m^2^hr when the feed water flow rate increases from 600 mL/min to 1200 mL/min. This represents about a 9.36% to 14.36% rise in flux for the PTFE membrane.

The general findings from Figure 7 and Figure 8 pointed to small performance variations in cooling plate materials with high thermal conductivities, including copper, aluminum, brass, and stainless steel. However, cooling plates with ultra-low thermal conductivities (acrylic and HDPE) performed poorly, with a huge performance discrepancy when compared to cooling plates with high thermal conductivities.

**Figure 9:**
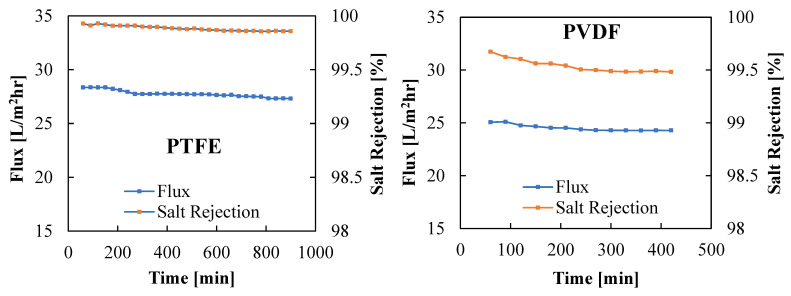
Variations of vapor flux and salt rejection factor with time for PTFE and PVDF membranes. Test Conditions: Coolant temperature = 15 °C, Coolant flow rate = 2000 mL/min, Feed Temperature = 70 °C, Feed flow rate = 1000 mL/min, and Brass cooling plate.

Figure 9 displays the variation in vapor flux and membrane salt rejection efficiency with time for the PTFE and PVDF membranes. The PVDF membrane was tested for 7 h, compared to 15 h for the PTFE membrane. A nearly stable vapor flux and salt rejection efficiency were observed when using the PTFE membrane, with both the flux and the salt rejection efficiency declining from 28.35 kg/m^2^hr to 27.31 kg/m^2^hr and 99.92% to 99.86%, respectively, after 15 h of continuous operation. The PVDF membrane recorded a lower flux and lesser salt rejection factor when compared to the PTFE membrane, with the PVDF membrane vapor flux decreasing from 25.06 kg/m^2^hr to 24.83 kg/m^2^hr and salt rejection efficiency deteriorating from 99.67% to 99.48%, respectively, after 7 h of continuous operation. Longer testing of the membrane may lead to further membrane degradation. The decline in vapor flux with time can be attributed to the concentration polarization as well as the effects of fouling and salt deposition on the membrane surface, as noticed in Figure 5 and Figure 6. The better salt rejection stability of the PTFE membrane may be attributed to its better hydrophobicity, as confirmed by the higher water contact angle reported in Table 1 and Figure 4b. The higher flux of the PTFE membrane may be due to its thinner active layer and higher contact angle, which encourage faster vapor transport across the membrane pores.

Table 4 shows the performance comparison between the current work and some other studies involving different MD membranes, MD modules, and cooling plates. The recorded vapor flux from the current study was relatively higher than those recorded using the AGMD module and quite comparable to those utilizing the WGMD or DCMD modules. However, the flux reported in [61] was far higher than the current flux due to the superior operating conditions and the adopted integrated SGMD-bubble column dehumidifier. The recorded GOR from this study falls within the previously registered GOR from other studies, including SGMD, AGMD, DCMD, and WGMD configurations.

## 4. Conclusions

A detailed investigation into the performance of the WGMD system for desalination of untreated real seawater with a TDS of 48,200 ppm was presented. The effect of using different cooling plate materials was examined and discussed. Furthermore, two commercial polymeric membranes of similar mean pore sizes were utilized in this study. The influence of key operating parameters, including the feed water temperature and feed water flow rate, was investigated against the vapor flux and gained output ratio. At all conditions, the PTFE membrane exhibited superior vapor flux and energy efficiency (GOR), with 9.36% to 14.36% higher flux when compared to the PVDF membrane at 0.6 to 1.2 L/min feed flow rate. Owing to its highest thermal conductivity, the copper plate attained the highest vapor flux, while the acrylic plate recorded the lowest vapor flux. The decreasing flux order of arrangement for different cooling plates is Cu > Al > Brass > SS > HDPE > Acrylic. The stainless-steel cooling plate registered the highest GOR, while the acrylic cooling plate obtained the lowest GOR. The increasing GOR order of arrangement for different cooling plates is Acrylic < HDPE < Cu < Al < Brass < SS. On the key operating variables, increasing the feed temperature increased the vapor flux almost exponentially (over 350% enhancement in flux when feed temperature increased from 40 to 70 °C) and improved the GOR in a decreasing trend. Whereas increasing the feed water flow rate enhanced the vapor flux and deteriorated the system GOR. Due to its superior hydrophobicity, the PTFE membrane also displayed better salt rejection stability in long-term tests. The performance variations in high thermally conducting cooling plates were meager while cooling plates with ultra-low thermal conductivities performed poorly and experienced a huge performance discrepancy in comparison to cooling plates with high thermal conductivities. In general, it is recommended to use copper as a cooling plate material for better system performance. On the other hand, cooling plate material with a very low thermal conductivity, such as a low-conducting polymer, should be avoided.

## Figures and Tables

**Table 1:** Summarized properties of the used membrane.

Properties		PTFE	PVDF
Active layer thickness		8 ± 2 μm	105 ± 3 μm
Support layer thickness		143 ± 16 μm	-
Total thickness		156 ± 14 μm	105 ± 3 μm
Pore Size	Mean	0.45 μm	0.50 μm
	Min.	0.40 μm	0.43 μm
	Max.	0.50 μm	0.57 μm
Porosity		74.78%	75.35%
Water Contact Angle		111.8°	85.4°
Liquid Entry Pressure (LEP)		2.4 ± 0.1 bar	0.4 ± 0.1 bar
Effective Area		7.316 × 10^−4^ m^2^	

**Table 2:** Cooling plates' thermal conductivity [56,57] and thickness.

Materials	Copper (Cu)	Aluminum (Al)	Brass	Stainless Steel (SS)	HDPE	Acrylic
Thermal Conductivity (W/m-K)	397 [57]	239 [56]	126 [57]	25 [56]	0.38–0.51 [57]	0.20 [56]
Thickness (mm)	2	2	2	2	2	2.3

**Table 3:** Gap temperature at different cooling plates, membranes, feed flow rates, and feed temperatures.

Feed Temperature [°C]	PTFE Membrane						PVDF Membrane					
	Cu	Al	Brass	SS	HDPE	Acrylic	Cu	Al	Brass	SS	HDPE	Acrylic
40	22.3	23.7	24.4	25.7	31.7	32.1	22.1	23.1	23.9	25.0	30.9	31.5
50	26.1	27.0	27.9	28.2	40.8	41.6	25.7	26.2	27.1	27.7	39.6	40.1
60	28.7	29.5	30.6	31.8	49.0	49.9	28.0	29.0	29.9	30.7	48.3	49.0
70	32.5	34.0	35.3	37.1	61.3	62.2	32.0	33.3	34.6	36.4	61.0	61.6
Feed Flow rate [mL/min]	PTFE Membrane						PVDF Membrane					
	Cu	Al	Brass	SS	HDPE	Acrylic	Cu	Al	Brass	SS	HDPE	Acrylic
600	31.9	33.2	34.6	36.3	60.1	60.9	31.1	32.4	33.8	35.3	59.9	60.3
800	32.2	33.7	35.0	36.6	60.8	61.6	31.6	32.8	34.2	35.8	60.5	61.1
1000	32.5	34.0	35.3	37.1	61.3	62.2	32.0	33.3	34.6	36.4	61.0	61.6
1200	32.9	34.3	35.9	37.7	61.8	62.9	32.4	33.8	35.0	37.1	61.6	62.0

**Table 4:** Performance comparison of various MD modules, membranes, and cooling plates.

Module Type/Cold Plate	Membrane Type	Conditions	Feed Salinity	Vapor Flux (kg/m^2^hr)	GOR (-)	References
Plate–Frame SGMD—bubble column dehumidifier/NA	PTFE	Feed temperature of 50 to 80 °C. Feed flow rate of 2.38 to 4.85 L/min. Coolant temperature of 15 °C. Coolant flow rate of 2.3 L/min.	2 g/L	60	0.76	[61]
Circular Plate–Frame AGMD module/NA	PP, PVDF, and PTFE	Feed temperature of 50 °C. Cold temperature of 7 °C	NA	7.6	1.01	[62]
Plate–Frame AGMD/NA	PTFE	Feed temperature of 40–85 °C. Feed flow rate = Cold flow rate = 20 L/min.	1 g/L and 35 g/L	6.5	0.28–0.79	[63]
Spiral wound AGMD/NA	PTFE	Feed temperature of 55–95 °C. Feed flow rate:1000 to 1300 L/h.	Untreated real seawater (55,000 μS/cm)	1.5	0.4–0.7	[64]
Tubular helical AGMD/copper	PTFE	Feed temperature of 45 to 75 °C. Feed flow rate = Coolant flow rate = 3 L/min.	20 g/L	20	0.11	[65]
Plate–Frame AGMD/copper	PVDF	Feed temperature of 20 to 85 °C. Gap width of 1.55 mm. Feed flow rate of 1 kg/s	NA	0.4	0.25	[66]
Plate–Frame AGMD/NA	PVDF and PVDF/PVP	Feed temperature of 40 to 80 °C. Feed flow rate of 1 to 2.5 L/min. Air gap width of 4, 6, 8, and 10 mm.	0.021 g/L and 35 g/L	8.734	NA	[67]
Plate–Frame WGMD, AGMD, DCMD/Metal	PVDF/PMMA	Feed temperature of 50–70 °C. Feed flow rate of 0.4–0.8 L/min. Coolant temperature of 20 °C. Coolant flow rate: 0.6 L/min.	35 g/L	26.04	0.653	[68]
Plate–Frame AGMD, WGMD/Stainless steel	SMM and PEI composite	Feed temperature of 35 to 80 °C	12 g/L and 30 g/L	16	NA	[43]
AGMD and WGMD/polymeric film	PTFE with PP support	Feed temperature of 80 °C. 17–20 °C coolant temperature.	35 g/L	12	<1 to 3.3	[69]
Plate–Frame WGMD/Copper, Aluminum, Brass, Stainless steel, HDPE, and Acrylic	PTFE with PP support and PVDF with PET substrate	Feed temperature of 50–70 °C. Feed flow rate of 0.6–1.2 L/min. Coolant temperature of 15 °C. Coolant flow rate: 2.0 L/min.	Untreated real seawater of 48.2 g/L	33.01	0.429	Present Study

PP = Polypropylene; PVP = Polyvinylpyrrolidone; PMMA = Poly (methyl methacrylate); SMM = Surface modifying macromolecule; PEI = Polyetherimide; PET = Polyethylene terephthalate.

## Data Availability

Not applicable.

## References

[1] Hu Y., Cheng H. (2013). Water pollution during China’s industrial transition. Environ. Dev..

[2] El-Dessouky H.T., Ettouney H.M. (1999). Multiple-effect evaporation desalination systems: Thermal analysis. Desalination.

[3] Ameri M., Mohammadi S.S., Hosseini M., Seifi M. (2009). Effect of design parameters on multi-effect desalinationsystem specifications. Desalination.

[4] Alhazmy M.M. (2011). Multi stage flash desalination plant with brine–feed mixing and cooling. Energy.

[5] Al-Obaidi M.A., Rasn K.H., Aladwani S.H., Kadhom M., Mujtaba I.M. (2022). Flexible design and operation of multi-stage reverse osmosis desalination process for producing different grades of water with maintenance and cleaning opportunity. Chem. Eng. Res. Des..

[6] Bahar R., Hawlader M.N.A., Woei L.S. (2004). Performance evaluation of a mechanical vapor compression desalination system. Desalination.

[7] Hamed O.A., Zamamiri A.M., Aly S., Lior N. (1996). Thermal performance and exergy analysis of a thermal vapor compression desalination system. Energy Convers. Manag..

[8] Hussain Soomro S., Santosh R., Bak C.U., Yoo C.H., Kim W.S., Kim Y.D. (2022). Effect of humidifier characteristics on performance of a small-scale humidification-dehumidification desalination system. Appl. Therm. Eng..

[9] Gude V.G. (2018). Emerging Technologies for Sustainable Desalination Handbook.

[10] Bin Abid M., Wahab R.A., Salam M.A., Gzara L., Moujdin I.A. (2023). Desalination technologies, membrane distillation, and electrospinning, an overview. Heliyon.

[11] Yan Z., Jiang Y., Liu L., Li Z., Chen X., Xia M., Fan G., Ding A. (2021). Membrane Distillation for Wastewater Treatment: A Mini Review. Water.

[12] Alkhudhiri A., Darwish N., Hilal N. (2012). Membrane distillation: A comprehensive review. Desalination.

[13] Islam M.R., Lin B., Yu Y., Chen C.-C., Malmali M. (2023). Comparative Energetics of Various Membrane Distillation Configurations and Guidelines for Design and Operation. Membranes.

[14] Ho C.D., Chen L., Yang Y.L., Chen S.T., Lim J.W., Chen Z.Z. (2023). Permeate Flux Enhancement in Air Gap Membrane Distillation Modules with Inserting Λ-Ribs Carbon-Fiber Open Slots. Membranes.

[15] Khan A., Ibrar I., Mirdad A., Al-Juboori R.A., Deka P., Subbiah S., Altaee A. (2023). Novel Approach to Landfill Wastewater Treatment Fouling Mitigation: Air Gap Membrane Distillation with Tin Sulfide-Coated PTFE Membrane. Membranes.

[16] Wu Z., Guo F. (2023). Finned Tubular Air Gap Membrane Distillation. Membranes.

[17] Alsalhy Q.F., Ibrahim S.S., Hashim F.A. (2018). Experimental and theoretical investigation of air gap membrane distillation process for water desalination. Chem. Eng. Res. Des..

[18] Fawzy M.K., Varela-Corredor F., Boi C., Bandini S. (2022). The Role of the Morphological Characterization of Multilayer Hydrophobized Ceramic Membranes on the Prediction of Sweeping Gas Membrane Distillation Performances. Membranes.

[19] Said I.A., Chomiak T., Floyd J., Li Q. (2020). Sweeping gas membrane distillation (SGMD) for wastewater treatment, concentration, and desalination: A comprehensive review. Chem. Eng. Process. Process. Intensif..

[20] Khayet M., Cojocaru C., Baroudi A. (2012). Modeling and optimization of sweeping gas membrane distillation. Desalination.

[21] Zhao S., Feron P.H.M., Xie Z., Zhang J., Hoang M. (2014). Condensation studies in membrane evaporation and sweeping gas membrane distillation. J. Membr. Sci..

[22] Abejón R., Saidani H., Deratani A., Richard C., Sánchez-Marcano J. (2019). Concentration of 1,3-dimethyl-2-imidazolidinone in Aqueous Solutions by Sweeping Gas Membrane Distillation: From Bench to Industrial Scale. Membranes.

[23] Hussein S.S., Ibrahim S.S., Toma M.A., Alsalhy Q.F., Drioli E. (2020). Novel chemical modification of polyvinyl chloride membrane by free radical graft copolymerization for direct contact membrane distillation (DCMD) application. J. Membr. Sci..

[24] Aljumaily M.M., Alayan H.M., Mohammed A.A., Alsaadi M.A., Alsalhy Q.F., Figoli A., Criscuoli A. (2022). The influence of coating super-hydrophobic carbon nanomaterials on the performance of membrane distillation. Appl. Water Sci..

[25] Hassan M.K., Khraisheh M., Tewodros B.N., Yang D.R., Park K. (2022). Design Parameters of a Direct Contact Membrane Distillation and a Case Study of Its Applicability to Low-Grade Waste Energy. Membranes.

[26] Khatri M., Francis L., Hilal N. (2023). Modified Electrospun Membranes Using Different Nanomaterials for Membrane Distillation. Membranes.

[27] Tetteh K., Rathilal S., Femi Bakare B., Almeshaal M.A., Choubani K. (2023). Using the Log Mean Temperature Difference (LMTD) and ε-NTU Methods to Analyze Heat and Mass Transfer in Direct Contact Membrane Distillation. Membranes.

[28] Nassif A.G., Ibrahim S.S., Majdi H.S., Alsalhy Q.F. (2022). Ethanol Separation from an Ethanol–Water Solution Using Vacuum Membrane Distillation. Membranes.

[29] Idrees H., Ali S., Sajid M., Rashid M., Khawaja F.I., Ali Z., Anwar M.N. (2023). Techno-Economic Analysis of Vacuum Membrane Distillation for Seawater Desalination. Membranes.

[30] Lu K.J., Zuo J., Chang J., Kuan H.N., Chung T.S. (2018). Omniphobic Hollow-Fiber Membranes for Vacuum Membrane Distillation. Environ. Sci. Technol..

[31] Abu-Zeid M.A.E.R., Zhang Y., Dong H., Zhang L., Chen H.L., Hou L. (2015). A comprehensive review of vacuum membrane distillation technique. Desalination.

[32] Jung W., Choe Y., Kim T., Ok J.G., Lee H.H., Kim Y.H. (2021). High-permeability vacuum membrane distillation utilizing mechanically compressed carbon nanotube membranes. RSC Adv..

[33] Elbessomy M.O., Elsamni O.A., Elsheniti M.B., Elsherbiny S.M. (2023). Optimum configurations of a compact hollow-fiber water gap membrane distillation module for ultra-low waste heat applications. Chem. Eng. Res. Des..

[34] Im B.G., Francis L., Santosh R., Kim W.S., Ghaffour N., Kim Y.D. (2022). Comprehensive insights into performance of water gap and air gap membrane distillation modules using hollow fiber membranes. Desalination.

[35] Memon S., Im B.G., Lee H.S., Kim Y.D. (2023). Comprehensive experimental and theoretical studies on material-gap and water-gap membrane distillation using composite membranes. J. Memb. Sci..

[36] Gao L., Zhang J., Gray S., Li J.-D. (2017). Experimental study of hollow fiber permeate gap membrane distillation and its performance comparison with DCMD and SGMD. Sep. Purif. Technol..

[37] Cheng L., Zhao Y., Li P., Li W., Wang F. (2018). Comparative study of air gap and permeate gap membrane distillation using internal heat recovery hollow fiber membrane module. Desalination.

[38] Yazgan-Birgi P., Hassan Ali M.I., Swaminathan J., Lienhard J.H., Arafat H.A. (2018). Computational fluid dynamics modeling for performance assessment of permeate gap membrane distillation. J. Memb. Sci..

[39] Lawal D.U. (2022). Performance enhancement of permeate gap membrane distillation system augmented with impeller. Sustain. Energy Technol. Assess..

[40] Ugrozov V.V., Elkina I.B., Nikulin V.N., Kataeva L.I. (2003). Theoretical and experimental research of liquid-gap membrane distillation process in membrane module. Desalination.

[41] Im B.G., Lee J.G., Kim Y.D., Kim W.S. (2018). Theoretical modeling and simulation of AGMD and LGMD desalination processes using a composite membrane. J. Membr. Sci..

[42] Yue C., Peng Y., Chen L., Schaefer L.A. (2021). Thermal analysis of a heat pump-based liquid gap membrane distillation H_2_SO_4_ system. Chem. Eng. Process. Process. Intensif..

[43] Essalhi M., Khayet M. (2014). Application of a porous composite hydrophobic/hydrophilic membrane in desalination by air gap and liquid gap membrane distillation: A comparative study. Sep. Purif. Technol..

[44] Francis L., Ghaffour N., Alsaadi A.A., Amy G.L. (2013). Material gap membrane distillation: A new design for water vapor flux enhancement. J. Membr. Sci..

[45] Cai J., Yin H., Guo F. (2020). Transport analysis of material gap membrane distillation desalination processes. Desalination.

[46] Swaminathan J., Chung H.W., Warsinger D.M., AlMarzooqi F.A., Arafat H.A., Lienhard J.H. (2016). Energy efficiency of permeate gap and novel conductive gap membrane distillation. J. Membr. Sci..

[47] Khalifa A.E. (2015). Water and air gap membrane distillation for water desalination—An experimental comparative study. Sep. Purif. Technol..

[48] Alawad S.M., Lawal D.U., Khalifa A.E., Aljundi I.H., Antar M.A., Baroud T.N. (2023). Analysis of water gap membrane distillation process with an internal gap circulation propeller. Desalination.

[49] Khalifa A.E. (2020). Flux enhanced water gap membrane distillation process-circulation of gap water. Sep. Purif. Technol..

[50] Alawad S.M., Khalifa A.E., Antar M.A. (2021). Performance analysis of multistage water gap membrane distillation system with economic evaluation. Appl. Therm. Eng..

[51] Ruiz-Aguirre A., Andrés-Mañas J.A., Fernández-Sevilla J.M., Zaragoza G. (2017). Modeling and optimization of a commercial permeate gap spiral wound membrane distillation module for seawater desalination. Desalination.

[52] Gao L., Zhang J., Gray S., Li J.-D. (2019). Modelling mass and heat transfers of Permeate Gap Membrane Distillation using hollow fibre membrane. Desalination.

[53] Gu M., Zhang J., Wang X., Tao H., Ge L. (2006). Formation of poly(vinylidene fluoride) (PVDF) membranes via thermally induced phase separation. Desalination.

[54] Smolders K., Franken A.C.M. (1989). Terminology for Membrane Distillation. Desalination.

[55] Lawal D., Abdul Azeem M., Khalifa A., Falath W., Baroud T., Antar M. (2022). Performance improvement of an air gap membrane distillation process with rotating fan. Appl. Therm. Eng..

[56] Carvill J. (1993). Mechanical Engineer’s Data Handbook.

[57] Martienssen W.W.H. (2005). . Springer Handbook of Condensed Matter and Materials Data.

[58] Mohd Ramli M.R., Ahmad A.L., Leo C.P. (2021). Surface Modification of Polytetrafluoroethylene Hollow Fiber Membrane for Direct Contact Membrane Distillation through Low-Density Polyethylene Solution Coating. ACS Omega.

[59] Boudenne A., Ibos L., Gehin E., Candau Y. (2003). A simultaneous characterization of thermal conductivity and diffusivity of polymer materials by a periodic method. J. Phys. D Appl. Phys..

[60] Zhou Y., Chen L., Huang M., Hu W., Chen G., Wu B. (2023). Experimental Investigation of the Desalination Process for Direct Contact Membrane Distillation Using Plate and Frame Membrane Module. Appl. Sci..

[61] Szczerbińska J., Kujawski W., Arszyńska J.M., Kujawa J. (2017). Assessment of air-gap membrane distillation with hydrophobic porous membranes utilized for damaged paintings humidification. J. Membr. Sci..

[62] Hussein A., Khalifa A.E., Alawad S.M., Antar M.A. (2022). Experimental investigation of membrane distillation with bubble column dehumidifier system for water desalination. Case Stud. Therm. Eng..

[63] Guillén-Burrieza E., Blanco J., Zaragoza G., Alarcón D.C., Palenzuela P., Ibarra M., Gernjak W. (2011). Experimental analysis of an air gap membrane distillation solar desalination pilot system. J. Memb. Sci..

[64] Banat F., Jwaied N., Rommel M., Koschikowski J., Wieghaus M. (2007). Performance evaluation of the “large SMADES” autonomous desalination solar-driven membrane distillation plant in Aqaba, Jordan. Desalination.

[65] Shahu V.T., Thombre S.B. (2022). Theoretical analysis and parametric investigation of an innovative helical air gap membrane desalination system. Appl. Water Sci..

[66] Summers E.K., Lienhard J.H. (2013). Experimental study of thermal performance in air gap membrane distillation systems, including the direct solar heating of membranes. Desalination.

[67] Gopi G., Vasanthkumar M., Arthanareeswaran G., Ismail A.F., Thuyavan Y.L., Goh P.S., Matsuura T. (2023). Performance, energy and economic investigation of airgap membrane distillation system: An experimental and numerical investigation. Desalination.

[68] Lin Y.X., Liou Y.K., Lee S.L., Chen S.Y., Tao F.T., Cheng T.W., Tung K.L. (2023). Preparation of PVDF/PMMA composite membrane with green solvent for seawater desalination by gap membrane distillation. J. Memb. Sci..

[69] Cipollina A., Di Sparti M.G., Tamburini A., Micale G. (2012). Development of a Membrane Distillation module for solar energy seawater desalination. Chem. Eng. Res. Des..

